# COX-2 metabolic products, the prostaglandin I_2_ and F_2α_, mediate the effects of TNF-α and Zn^2+^ in stimulating the phosphorylation of Tau

**DOI:** 10.18632/oncotarget.21853

**Published:** 2017-10-16

**Authors:** Yue Wang, Pei-Pei Guan, Xin Yu, Yan-Su Guo, Ying-Jie Zhang, Zhan-You Wang, Pu Wang

**Affiliations:** ^1^ College of Life and Health Sciences, Northeastern University, Shenyang, P.R. China; ^2^ Department of Tissue Culture, Liaoning University of Traditional Chinese Medicine, Shenyang, P.R. China; ^3^ Key laboratory of Hebei Neurology, Hebei Medical University, Shijiazhuang, P.R. China; ^4^ Institute of Cardiocerebrovascular Disease, Hebei Medical University, Shijiazhuang, P.R. China; ^5^ College of Biology, Hunan University, Changsha, P.R. China; ^6^ Shenzhen Institute, Hunan University, Shenzhen, P.R. China

**Keywords:** cyclooxygenase-2, prostaglandins, tumor necrosis factor α, zinc transporter 3, tau phosphorylation

## Abstract

Although the roles of cyclooxygenase-2 (COX-2) and prostaglandins (PGs) in regulating amyloid precursor protein (APP) cleavage and β-amyloid protein (Aβ) production have been the subjects of numerous investigations, their effects on tau phosphorylation have been largely overlooked. Using human Tau^P301S^ transgenic (Tg) mice as *in vivo* model, our results demonstrated that PGI_2_ and PGF_2α_ mediated the effects of tumor necrosis factor α (TNF-α) and Zinc ions (Zn^2+^) on upregulating the phosphorylation of tau via the PI3-K/AKT, ERK1/2 and JNK/c-Jun signaling pathways. Specifically, we initially found that high level of Zn^2+^ upregulates the expression of COX-2 via stimulating the activity of TNF-α in a zinc transporter 3 (ZnT3)-dependent mechanism. COX-2 upregulation then stimulates the phosphorylation of tau at both Ser 202 and Ser 400/Thr 403/Ser 404 via PGI_2_ and F_2α_ treatment either in i.c.v.-injected mice or in n2a cells. Using n2a cells as *in vitro* model, we further revealed critical roles for the PI3-K/AKT, ERK1/2 and JNK/c-Jun pathways in mediating the effects of PGI_2_ and F_2α_ in the phosphorylation of tau. Finally, NS398 treatment delayed the onset of cognitive decline in Tau^P301S^ Tg mice according to the nest construction or limb clasping test.

## INTRODUCTION

Cyclooxygenase-2 (COX-2) was first found to be upregulated during the early stage of Alzheimer’s disease (AD) two decades ago [1]. Because AD is pathologically characterized by β-amyloid protein (Aβ) deposition in amyloid plaques (APs) and by tau phosphorylation in neurofibrillary tangles (NFTs) [2], numerous investigations have since been carried out to evaluate the roles of COX-2 and its metabolic products, prostaglandins (PGs), in Aβ deposition. However, the effects of COX-2 and PGs on tau phosphorylation were largely overlooked. To the best of our knowledge, COX-2 suppression by ibuprofen treatment decreases the phosphorylation of tau in APP/PS1/Tau Tg mice [3]. In addition, COX-2 overexpression induces the formation of NFTs in patients with Fukuyama-type congenital muscular dystrophy [4]. As the metabolic product of COX-2, 15d-PGJ_2_ treatment increases the cleavage of tau protein and results in the formation of ∆tau, which accelerates the formation of NFTs in human neuroblastoma SK-N-SH cells [5]. These observations clearly indicated that COX-2 and PGs might be able to regulate the phosphorylation of tau and the formation of NFTs during the development of AD.

Although we could not find more direct evidence showing the relationship between COX-2/PGs and tau phosphorylation, we cannot exclude the possibility that other molecules, such as cytokines, may induce tau phosphorylation via enhancing the expression of COX-2 and PGs. For example, in mice, TNF-α injection induces cognitive decline via increasing the expression of COX-2 [6]. In addition, IL-1β treatment has also been reported to impair the learning ability of mice via upregulating the production of PGE_2_ [7, 8]. Therefore, it is highly plausible that cytokines, including TNF-α, IL-1β and IL-6, can increase the phosphorylation of tau via COX-2 and PGs in different AD experimental models [9-14].

Related to the possible roles of cytokines in tau phosphorylation via COX-2 and PGs, it has recently been reported that Zn^2+^ dyshomeostasis can regulate cytokine expression. For instance, Chu *et al.* [15, 16] reported that TNF-α gene expression is increased following Zn^2+^ treatment in type 2 diabetes mellitus, Zn^2+^ treatment has ability to induce the expression of TNF-α in wistar rats [17]. In addition, the levels of Zn^2+^ and TNF-α have been reported as potential blood biomarkers for disease severity in the Taiwanese population with AD [18]. These observations also indicate that Zn^2+^ might be able to regulate tau phosphorylation. Notably, Zn^2+^ has been reported to be significantly increased within the cerebral cortex of AD patient [19, 20]. Moreover, tau hyperphosphorylation has been observed to be correlated with brain Zn^2+^ metabolism disorders [21]. Indeed, treatment with low concentrations of Zn^2+^ can induce the aggregation of tau protein *in vitro*. Mechanistically, previous studies revealed that Zn^2+^ induced tau hyperphosphorylation by activating the ERK1/2, JNK, GSK-3β, and p38 pathways [19]. Further, Zn^2+^ stimulated tau phosphorylation by inhibiting the protein phosphatase PP2A in cultured rat hippocampal slice [21]. In addition, the Zn^2+^ chelator clioquinol (CQ) significantly and reversibly reduced the phosphorylation of tau in rats. Actually, Zn^2+^ is not free to pass through the cell membrane and require specific transporters and membrane channels to participate in cell metabolism. The regulation of Zn^2+^ homeostasis is closely related to zinc transporter (Zinc transporter, ZnT) in brain. Specifically, ZnT3 is mainly distributed in the central nervous system, and located in synaptic vesicles of axon terminals in zinc glutamate neurons. Its function is to transport Zn^2+^ into in synaptic vesicles. It is also suggested that the abnormal expression and distribution of ZnT3 may be the main cause of the disorder of Zn^2+^ metabolism in AD brain [22]. Consistent with the critical roles of Zn^2+^ in tau phosphorylation, the abnormal expression and distribution of zinc transporter 3 (ZnT3) have also been reported to cause AD via disrupting Zn^2+^ metabolism in synaptic vesicles [23-26]. Furthermore, knockout of ZnT3 in APP mice significantly decreased the production of Aβ and formation of APs [27, 28].

Although all these studies indicated the involvement of COX-2, PGs, TNF-α and Zn^2+^ in regulating tau phosphorylation, the relationships and mechanisms linking these bioactive molecules remain unknown. Therefore, the purpose of the current study is to decipher the mechanisms of tau phosphorylation in Tau^P301S^ Tg mice. These Tau^P301S^ Tg mice express the P301S mutant form of human microtubule-associated protein tau (MAPT). At three months of age, transgenic mice exhibit clasping and limb retraction when lifted by the tail, which progresses to limb weakness. By ten months of age, the mice exhibit a hunched back and paralysis, followed by inability to feed. The onset of neurofibrillay tangles (NFTs) formation in the neocortex, amygdala, hippocampus, brain stem and spinal cord is five months of age. Transgenic mice display neuroinflammation with microglial activation and astrogliosis. The ultrastructure of the NFTs-like lesions detected is similar to that found in brain lesions of human Alzheimer’s disease patients. Specifically, our data demonstrated that the production of PGI_2_/PGF_2α_ and the expression of COX-2 were induced by Zn^2+^ via the TNF-α pathway in a ZnT3-dependent manner. In addition, high levels of PGI_2_ and F_2α_ stimulated the phosphorylation of tau at both Ser 202 and Ser 400/Thr 403/Ser 404 via the PI3-K/AKT, ERK1/2 and JNK/c-Jun pathways. These observations provide novel insights into the mechanisms of tau phosphorylation and may be instrumental for identifying therapeutic targets for combating AD.

## RESULTS

### The expression of COX-2, TNF-α, ZnT3 and phosphorylation of tau at the sites of both Ser 202 and Ser 400/Thr 403/Ser 404 were elevated in 6-month-old Tau^P301S^ Tg mice

**Figure 1:**
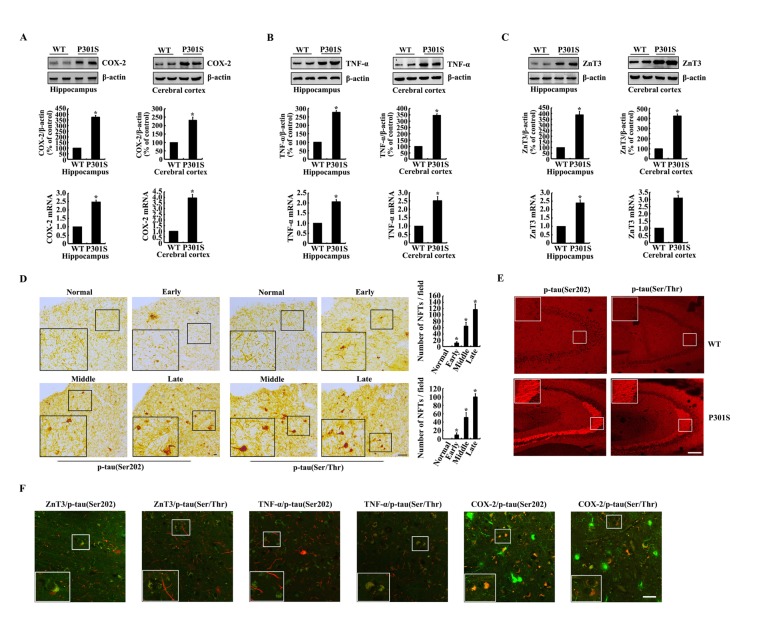
The expression of COX-2, TNF-α, ZnT3 and phosphorylation of tau at the sites of both Ser 202 and Ser 400/Thr 403/Ser 404 were upregulated in 6-month-old Tau^P301S^ Tg mice (**A**-**C**, **E**) The brains of WT or Tau^P301S^ Tg mice at the age of 6-month-old were collected following anesthesia and perfusion (n=8). (**A**-**C**) The mRNA and protein expression of COX-2, TNF-α and ZnT3 in the hippocampus and cerebral cortex of WT and Tg mice was determined by western blots. (**E**) The immunoreactivity of p-tau was determined by immunohistochemistry using an anti-tau (Ser202) or anti-tau (Ser400/Thr403/Ser404) antibody. (**D**, **F**) The tissue blocks of human brains at different stages of AD were collected by the New York Brain Bank at Columbia University. Free-floating slices (40 µm) were prepared by flat slicer (n=3). (**D**) The phosphorylation of tau at the sites of both Ser 202 and Ser 400/Thr 403/Ser 404 were immunostained by immunohistochemistry using specific antibodies. (**F**) The slices of AD patients were double stained with p-tau and ZnT3, TNF-α and COX-2, respectively. The data represent the means ± S.E. *, *p<0.05* with respect to WT controls.

In view of the possible roles of COX-2, TNF-α and ZnT3 in tau phosphorylation, we initially determined the expression of COX-2, TNF-α and ZnT3 in 6-month-old Tau^P301S^ Tg mice. As shown in Figure 1A-1C, the mRNA and protein expression of COX-2, TNF-α and ZnT3 were elevated in 6-months-old Tau^P301S^ Transgenic (Tg) mice compared to the paired wild type (WT) controls. In addition, immunoreactivity of p-Tau (Ser 202 and Ser 400/Thr 403/Ser 404) was progressively upregulated during the course of AD development (Figure 1D). Of note, the phosphorylated tau protein concentrated in the NFTs of late stage of AD patients, and the number of NFTs was increased gradually as the disease progresses (Figure 1D). As the limited accessibility of human samples from AD patients, we further found that the phosphorylation of tau at the sites of both Ser 202 and Ser 400/Thr 403/Ser 404 was increased in the CA3 region of 6-month-old Tau^P301S^ Tg mice compared to that of the paired WT controls (Figure 1E). To further reveal the relationship of these molecules, we double stained the brains of AD patients with ZnT3, TNF-α, COX-2 and phosphorylated tau. The results demonstrated that phosphorylated tau colocalized with ZnT3, TNF-α and COX-2, respectively (Figure 1F). Collectively, these observations indicate the internal relationship among these molecules, which result in AD development and progression.

### Critical roles of Zn^2+^ and TNF-α in upregulating the expression of COX-2 in mice.

**Figure 2:**
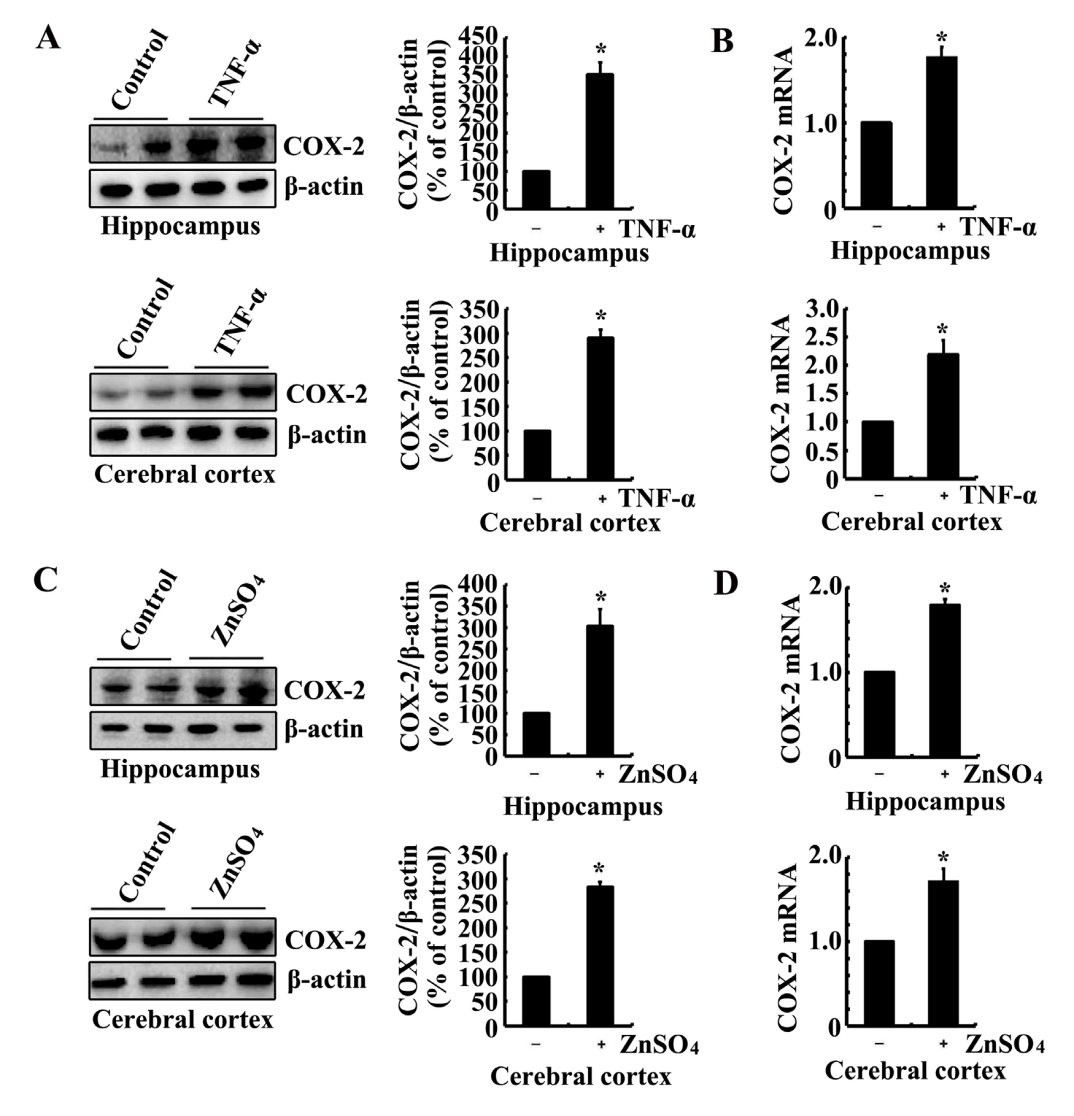
Intracerebroventricular injection of ZnSO_4_ and TNF-α to the ventricles of WT mice increases the expression of COX-2 (**A**-**D**) The WT mice at the age of 6 months were injected (i.c.v) with ZnSO_4_ (10 µg/5 µl) or TNF-α (1 ng/5 µl) (n=12). The brains were then collected after 24 h. mRNA and protein levels of COX-2 were determined by qRT-PCR and western blot, respectively. Total amounts of GAPDH and β-actin served as an internal control. The data represent the means ± S.E.. *, *p<0.05* with respect to PBS (-)-injected (i.c.v) controls.

**Figure 3:**
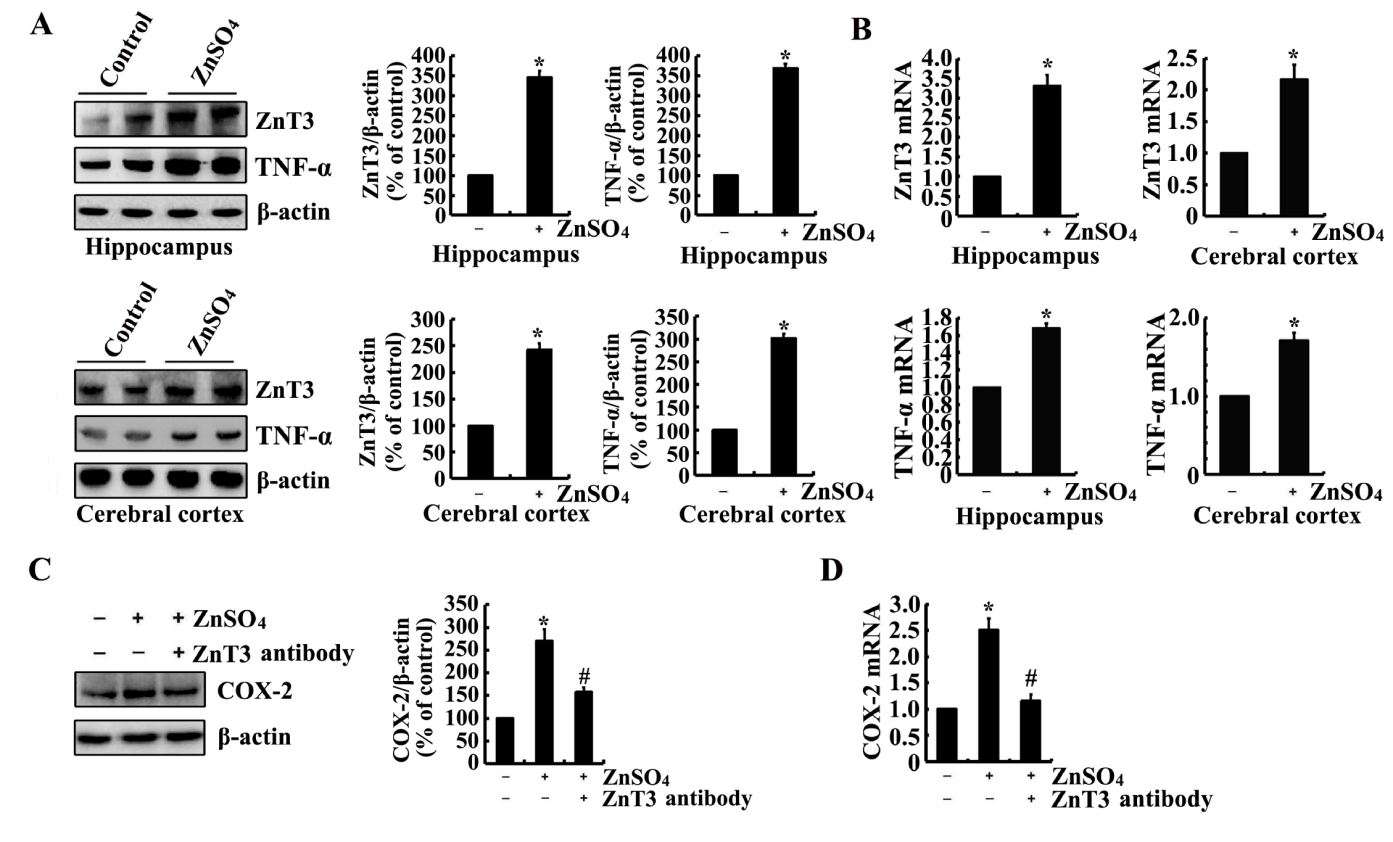
ZnSO_4_ stimulates the expression of COX-2 via inducing the expression of ZnT3 and TNF-α (**A**, **B**) The WT mice at the age of 6 months were injected (i.c.v.) with ZnSO_4_ (10 µg/5 µl) (n=12). The brains were then collected after 24 h. mRNA and protein levels of ZnT3 and TNF-α were determined by qRT-PCR and western blot, respectively. Total amounts of GAPDH and β-actin served as an internal control. (**C**-**D**) In select experiments, n2a cells were treated with ZnSO4 (10 µM) in the absence or presence of ZnT3 antibody (1 µg/ml). (**C**, **D**) mRNA and protein levels of COX-2 were determined by qRT-PCR and western blots, respectively. Total amounts of GAPDH and β-actin served as an internal control. The data represent the means ± S.E.. *, *p<0.05* with respect to PBS (-) or vehicle-treated controls. #, *p<0.05* with respect to ZnSO_4_-treated alone.

As the possible roles of Zn^2+^ and TNF-α in tau phosphorylation via COX-2, we determined the effects of Zn^2+^ and TNF-α on the expression of COX-2. To this purpose, we injected (i.c.v, intracerebroventricular injection) TNF-α (1 ng/5 µl) to the ventricles of 6-month-old WT mice. The results demonstrated that TNF-α injection (i.c.v) stimulated the mRNA and protein expression of COX-2 (Figure 2A, 2B). Similarly, ZnSO_4_ (10 µg/5 µl) injection (i.c.v) to the ventricles of 6-month-old WT mice induces the mRNA and protein expression of COX-2 in the hippocampus and cerebral cortex of 6-month-old WT mice (Figure 2C, 2D). More interestingly, ZnSO_4_ injection (i.c.v) clearly enhances the expression of ZnT3 or TNF-α in the hippocampus and cerebral cortex of 6-month-old WT mice (Figure 3A, 3B). When we blocked the biological function of ZnT3 by antibody, the expression of COX-2 was reduced in n2a cells (Figure 3C, 3D). These observations clearly indicated that ZnT3 mediated the effects of Zn^2+^ on inducing the expression of COX-2.

### Effects of PGI_2_ and F_2α_ on stimulating the phosphorylation of tau at the sites of both Ser 202 and Ser 400/Thr 403/Ser 404

**Figure 4:**
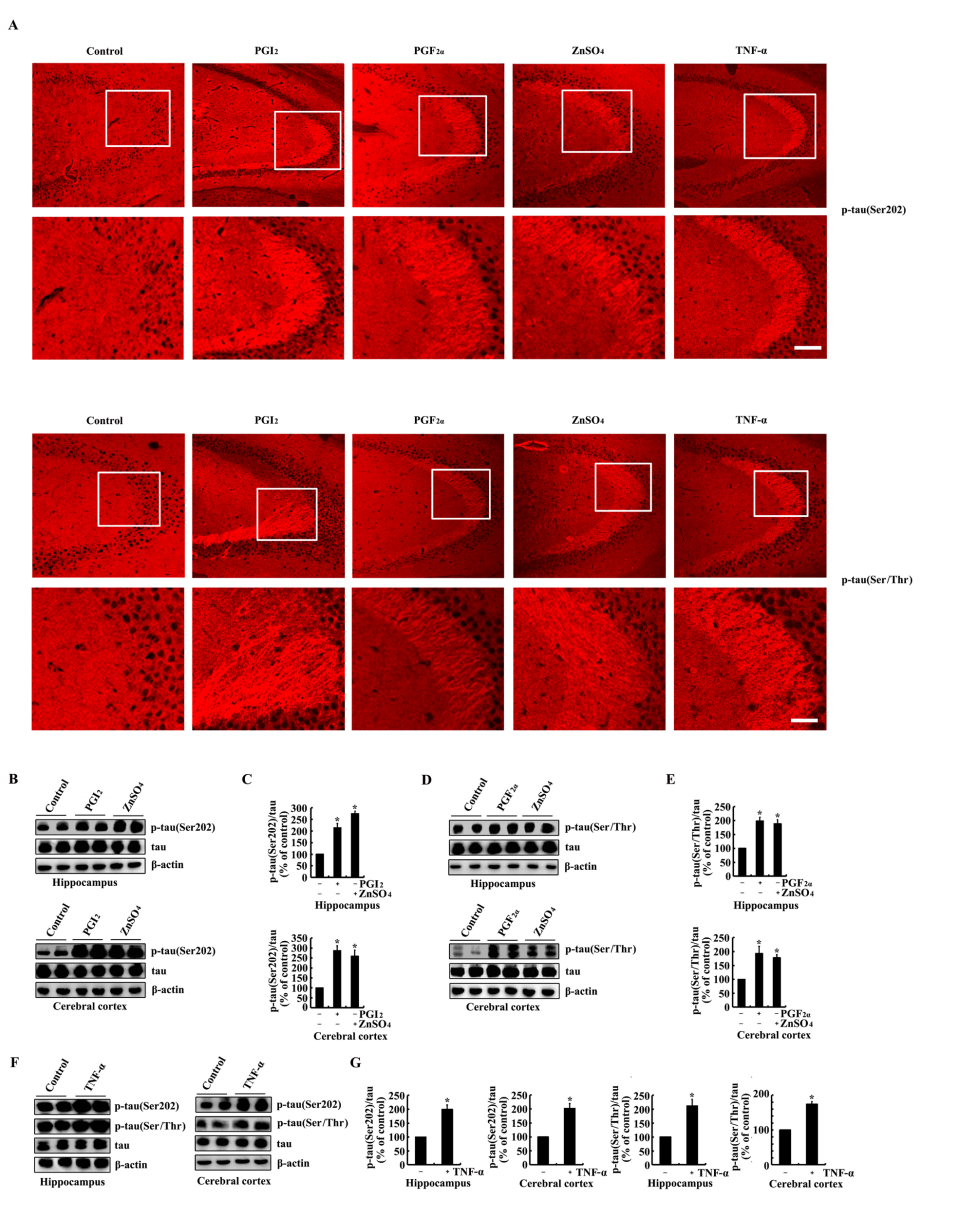
PGI_2_ and F_2α_ mediated the effects of ZnSO_4_ and TNF-α on stimulating the phosphorylation of tau at the sites of both Ser 202 and Ser 400/Thr 403/Ser 404 The WT mice at the age of 6 months were injected (i.c.v) with PGI_2_ (1 µg/5 µl), PGF_2α_ (1 µg/5 µl), ZnSO_4_ (10 µg/5 µl) or TNF-α (1 ng/5 µl) (n=12). (**A**) The phosphorylation of tau at the sites of both Ser 202 and Ser 400/Thr 403/Ser 404 were determined by immunostaining using the correspondent antibody. (**B**-**G**) Western blots were further used to determine the phosphorylation of tau at the sites of both Ser 202 and Ser 400/Thr 403/Ser 404. The intensity of bands was analyzed by Bio-Rad imaging software. The data represent the means ± S.E.. *, *p<0.05* with respect to PBS (-)-injected (i.c.v) controls.

As the metabolic products of COX-2, immunostaining experiments were carried out to determine the effects of PGI_2_ and F_2α_ on the phosphorylation of tau. As a consequence, PGI_2_ and F_2α_ injection (i.c.v) for 24 h to the ventricles of mice clearly increases the immunoreactivity of p-tau at the sites of both Ser 202 and Ser 400/Thr 403/Ser 404 (Figure 4A). To validate the critical roles of TNF-α and ZnSO_4_ in upregulating the expression of COX-2, we further determined the effects of TNF-α and ZnSO_4_ on the phosphorylation of tau. As expected, the results showed that injection (i.c.v) of TNF-α (1 ng/5 µl) and ZnSO_4_ (10 µg/5 µl) to the ventricles of mice obviously induced the phosphorylation of tau at the sites of Ser 202 and Ser 400/Thr 403/Ser 404 (Figure 4A). These immunostaining results were further validated by qRT-PCR and western blots. In detail, PGI_2_ and F_2α_ injection (i.c.v) were able to stimulate the phosphorylation of tau at the sites of both Ser 202 and Ser 400/Thr 403/Ser 404 in the himmpocampus and cerebral cortex of mice (Figure 4B-E). In addition, the phosphorylation of tau at the sites of Ser 202 and Ser 400/Thr 403/Ser 404 was also induced by the stimulation of ZnSO_4_ and TNF-α in the hippocampus and cerebral cortex of mice (Figure 4B-4G). Based on these observations, our data have verified the fact that PGI_2_ and F_2α_ potentially mediated the effects of COX-2 on inducing the phosphorylation of tau at the sites of Ser 202 and Ser 400/Thr 403/Ser 404 in mice, which in turn potentially contribute the effects of Zn^2+^ and TNF-α on the phosphorylation of tau.

### Key roles of COX-2 in mediating the effects of Zn^2+^ and TNF-α on stimulating the phosphorylation of tau at the sites of both Ser 202 andSer 400/Thr 403/Ser 404

**Figure 5:**
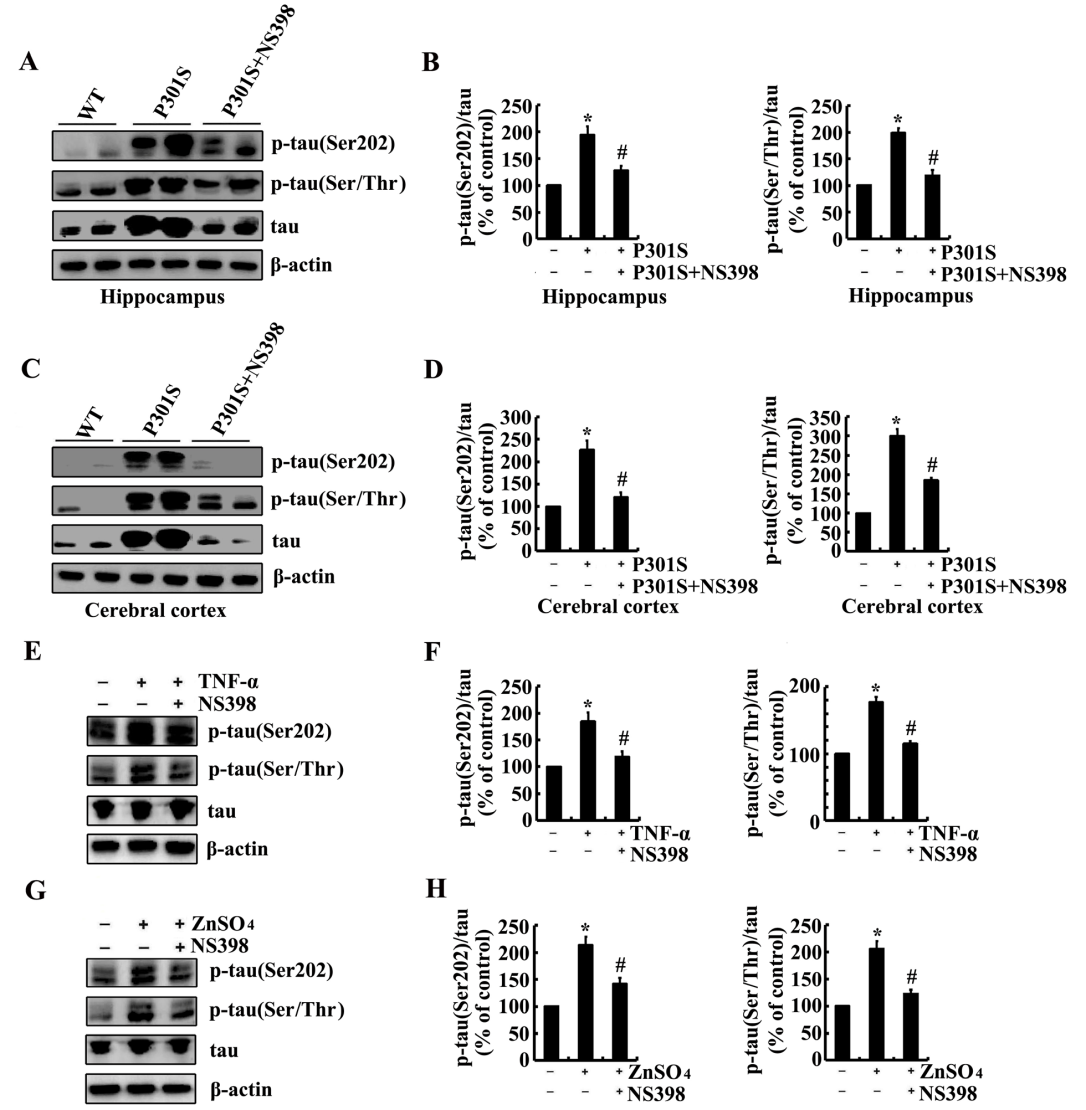
NS398 treatment decreases the phosphorylation of tau at the sites of Ser 202 and Ser 400/Thr 403/Ser 404 in 6-month-old Tau^P301S^ Tg mice or TNF-α- or ZnSO_4_-activated n2a cells (**A**-**D**) The Tau^P301S^ Tg mice at the age of 6 months were injected (i.c.v) with NS398 (1 µg/5 µl) and the brains were then collected after 24 h. (**E**-**H**) In select experiments, the n2a cells were treated with TNF-α (10 ng/ml) or ZnSO_4_ (10 µΜ) in the absence or presence of NS398 (100 µΜ) for 24 h. The phosphorylation of tau at the sites of both Ser 202 and Ser 400/Thr 403/Ser 404 were determined by western blots. The intensity of bands was analyzed by Bio-Rad imaging software. The data represent the means ± S.E. *, *p<0.05* with respect to WT or vehicle-treated controls. #, *p<0.05* with respect to Tau^P301S^ Tg mice or TNF-α-, ZnSO_4_-treated alone.

As the potential roles of Zn^2+^ and TNF-α in inducing the phosphorylation of tau via upregulating the expression of COX-2, it is necessary to verify if COX-2 mediates the effects of Zn^2+^ and TNF-α on stimulating the phosphorylation of tau. As a first step, experiments were carried out to inject (i.c.v) into the ventricles of 6-month-old Tau^P301S^ Tg mice. The results demonstrated that NS398, a COX-2 inhibitors (1 µg/5 µl) treatment clearly decreased the phosphorylation of tau at the sites of both Ser 202 and Ser 400/Thr 403/Ser 404 in the hippocampus and cerebral cortex of Tau^P301S^ Tg mice (Figure 5A-5D). In addition, NS398 (100 µM) treatment blocked the effects of TNF-α (10 ng/ml) and ZnSO_4_ (10 µM) on stimulating the phosphorylation of tau at the sites of Ser 202 and Ser 400/Thr 403/Ser 404 in n2a cells (Figure 5E-5H). Therefore, it is clear that Zn^2+^ and TNF-α regulate the phosphorylation of tau via COX-2 activation.

### Signaling pathways involved in regulating the phosphorylation of tau

**Figure 6:**
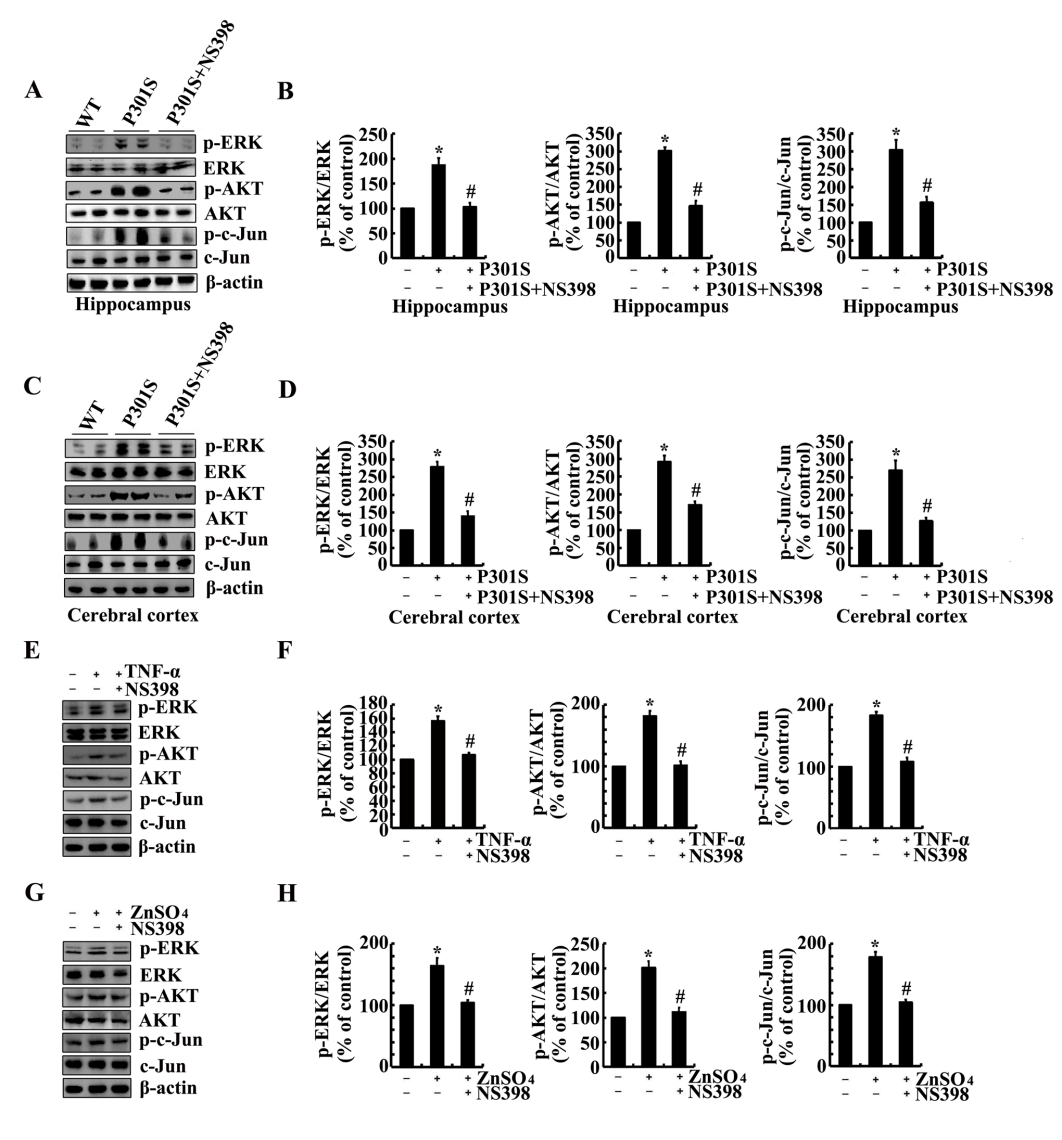
NS398 treatment decreases the activity of PI3-K/AKT, ERK1/2 and JNK/c-Jun pathways in 6-month-old Tau^P301S^ Tg mice and TNF-α- or ZnSO_4_-stimulated n2a cells (**A**-**D**) The Tau^P301S^ Tg mice at the age of 6 months were injected (i.c.v) with NS398 (1 µg/5 µl) and the brains were then collected after 24 h. (**E**-**H**) In select experiments, the n2a cells were treated with TNF-α (10 ng/ml) or ZnSO_4_ (10 µΜ) in the absence or presence of NS398 (100 µΜ) for 24 h. The phosphorylation of AKT, ERK1/2 and c-Jun were determined by western blots. The intensity of bands was analyzed by Bio-Rad imaging software. The data represent the means ± S.E. *, *p<0.05* with respect to WT or vehicle-treated controls. #, *p<0.05* with respect to Tau^P301S^ Tg mice or TNF-α-, ZnSO_4_-treated alone.

**Figure 7:**
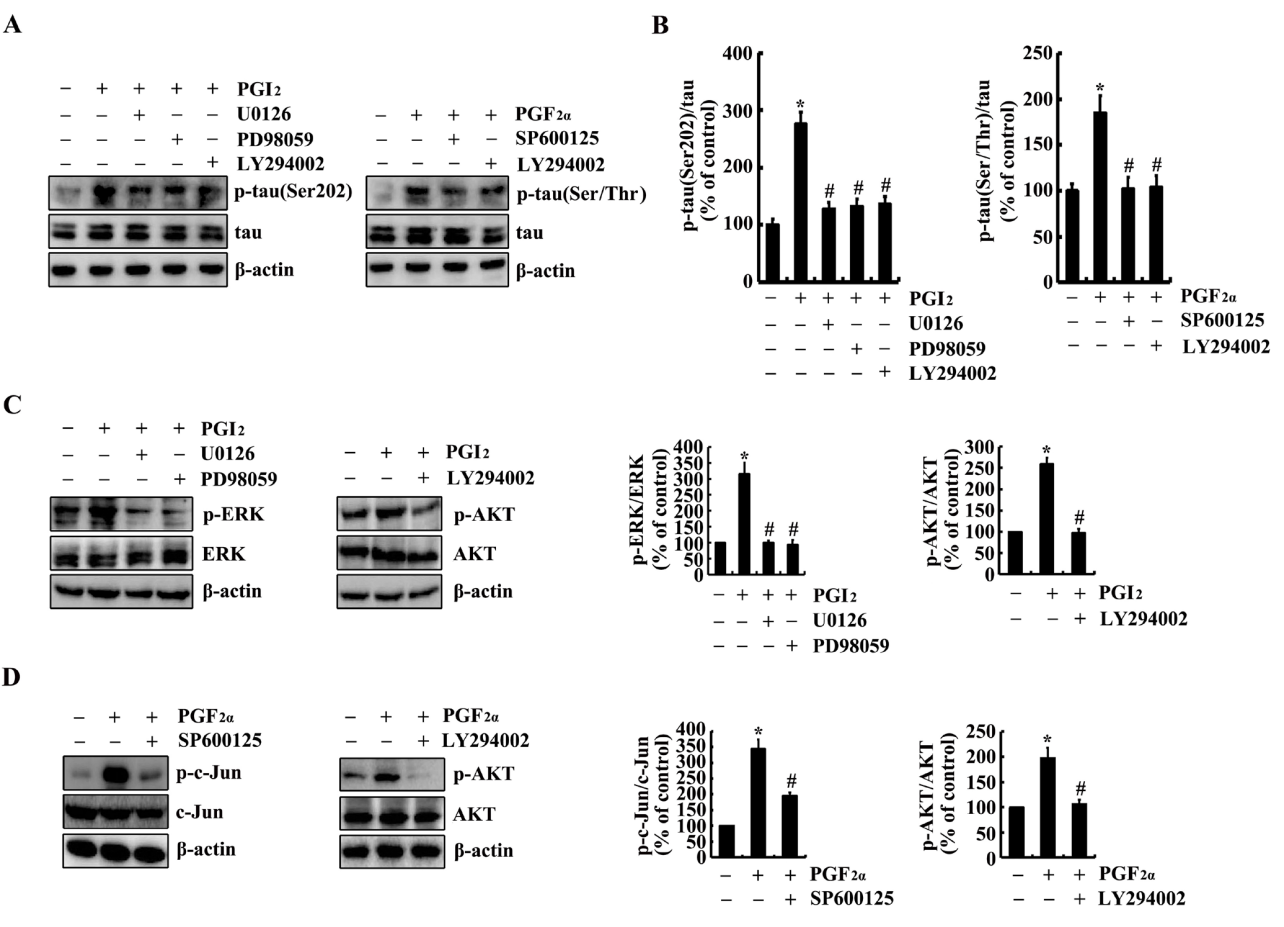
Involvement of PI3-K/AKT, ERK1/2 and JNK/c-Jun pathways in regulating tau phosphorylation at the sites of both Ser 202 and Ser 400/Thr 403/Ser 404 in PGI_2_- or PGF_2α_-stimulated n2a cells n2a cells were treated with PGI_2_ (10 µΜ) or PGF_2α_ (1 µΜ) in the absence or presence of PI3-K inhibitor, LY294002 (20 µΜ), ERK1/2 inhibitor, PD98059 (20 µΜ) and U0126(20 µΜ), JNK inhibitor, SP600125 (10 µΜ), for 24 h. (**A**, **B**) The phosphorylation of tau at the sites of both Ser 202 and Ser 400/Thr 403/Ser 404 were determined by western blots. (**C**, **D**) The phosphorylation of AKT, ERK1/2 and c-Jun were determined by western blots. The intensity of bands was analyzed by Bio-Rad imaging software. The data represent the means ± S.E. *, *p<0.05* with respect to vehicle-treated controls. #, *p<0.05* with respect to PGI_2_ or F_2α_-treated alone.

We next aimed to determine the possible signaling pathways in regulating the phosphorylation of tau. As a consequence, experiments were carried out to inject NS398 (1 µg/5 µl) to the ventricles of 6-month-old Tau^P301S^ Tg mice. The results demonstrated that the phosphorylation of ERK1/2, AKT and c-Jun were highly induced in 6-month-old Tau^P301S^ Tg mice (Figure 6A-6D). In addition, NS398 treatment significantly decreased the phosphorylation of ERK1/2, AKT and c-Jun in 6-month-old Tau^P301S^ Tg mice (Figure 6A-6D). In line with these *in vivo* results, we further treated n2a cells with TNF-α (10 ng/ml) and ZnSO_4_ (10 µM) in the absence or presence of NS398 (100 µM) for 24 h. The results demonstrated that NS398 clearly decreased the phosphorylation of ERK1/2, AKT and c-Jun in TNF-α- or ZnSO_4_-treated n2a cells (Figure 6E-6H). To further verify the involvement of these possible signaling pathways, we treated the n2a cells with PGI_2_ or PGF_2α_ in the absence or presence of inhibitors of signaling pathways, respectively. The results demonstrated that treatment with inhibitors of PI3-K/AKT, ERK1/2 and JNK/c-Jun obviously suppressed the PGI_2_- and F_2α_-activated tau phosphorylation (Figure 7A-7D).

### NS398 treatment decreases the cognitive decline of Tau^P301S^ Tg mice

**Figure 8:**
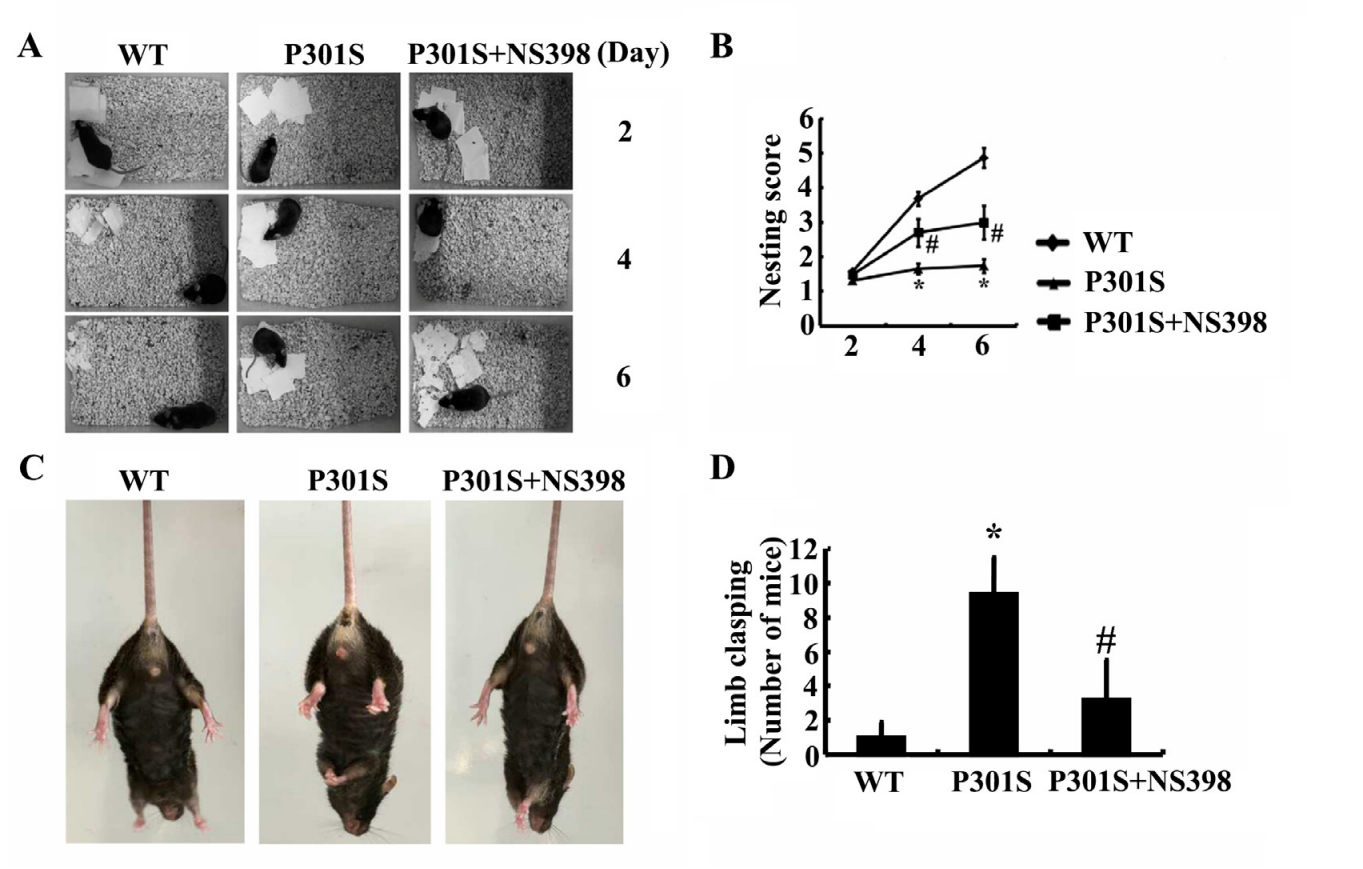
COX-2 plays pivotal roles in impairing the learning ability of Tau^P301S^ Tg mice (**A**, **B**) Tau^P301S^ Tg mice at the age of 1 months old were treated with NS398 (1 mg/kg/d) for 5 months (n=12). The learning ability of mice was then determined by nest construction. Baseline data were obtained on d 0, after the addition of paper towels in clean cages. Habituation behavior was recorded everyday and the nesting score was obtained as described in “Materials and Methods”. (**C**, **D**) Abnormal limb clasping of the mice were determined by lifting the tail. Each group was tested six times.

As the pivotal roles of COX-2 in regulating the phosphorylation of tau, the questions are easily raised if COX-2 overexpression will finally affect the learning ability of Tau^P301S^ Tg mice. In accordance with this hypothesis, nest construction assays were designed to determine the learning ability of mice [29]. As expected, the learning ability of Tau^P301S^ Tg mice appeared to be impaired at the age of 6-month-old (Figure 8A, 8B). In addition, NS398 treatment improves the ability of nest construction for 6-month-old Tau^P301S^ Tg mice (Figure 8A, 8B). To further verify these behavioral deficiencies, limb clasping experiments were carried out as to determine the hallmark of AD. The results demonstrated that the Tau^P301S^ Tg mice showed limb-clasping reflexes at 6 months old, which was partially reversed by NS398 treatment (Figure 8C, 8D). These observations further emphasize the pivotal roles of COX-2 in regulating the development or progression of AD.

**Figure 9:**
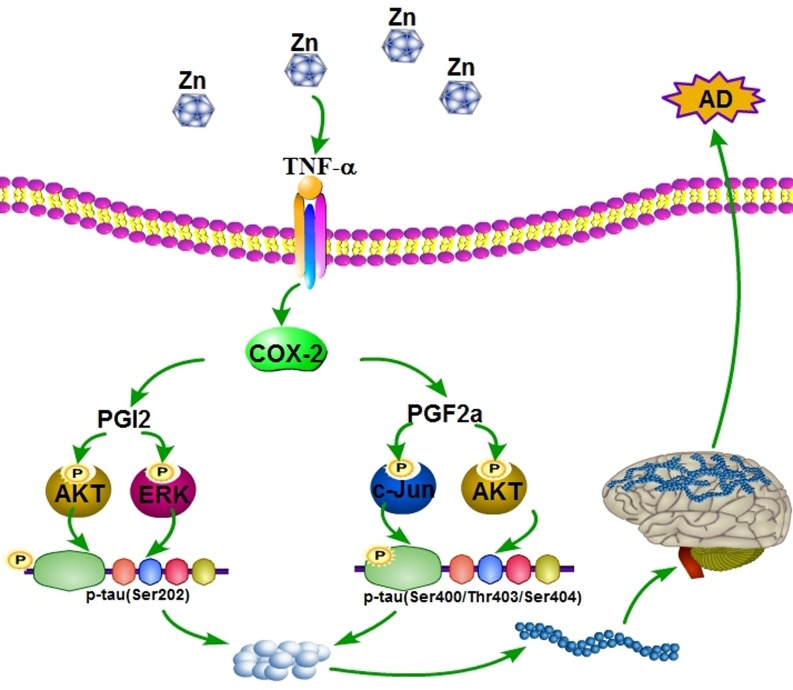
Proposed events of the signaling cascade regulate the pathogenesis of AD In detail, ZnSO_4_ have ability to upregulate the expression of COX-2 and the production of PGI_2_ and F_2α_ via inducing the expression of TNF-α. Highly accumulated PGI_2_ and F_2α_ induce the phosphorylation of tau at the sites of both Ser 202 and Ser 400/Thr 403/Ser 404 via PI3-K/AKT, ERK1/2 and JNK/c-Jun pathways. These actions finally result in the cognitive decline of Tau^P301S^ Tg mice.

In summary, our data revealed that ZnSO_4_ played critical effects on stimulating the expression of TNF-α in a ZnT3-dependent mechanism. Ηighly expressed TNF-α induced the expression of COX-2. COX-2 induced the phosphorylation of tau at the sites of both Ser 202 and Ser 400/Thr 403/Ser 404 via PGI_2_- and PGF_2α_-dependent PI3-K/AKT, ERK1/2 and JNK/c-Jun activation pathways. Finally, the phosphorylation of tau protein will impair the learning ability of Tau^P301S^ Tg mice (Figure 9).

## DISCUSSION

COX-2 and its metabolic products, prostaglandins (PGs), have been reported to be upregulated during the early stage of AD [30, 31]. Although the interest in investigating COX-2 was disrupted by the failure of clinical trials [32], the results of these clinical trials have been called into question based on the lack of a mechanistic study for guidance. Therefore, we investigated the mechanisms by which COX-2 metabolic products, PGI_2_ and F_2α_ might regulate the phosphorylation of tau. Specifically, TNF-α and ZnSO_4_ stimulated the expression of COX-2 and the production of PGs, such as PGI_2_ and F_2α_. Notably, ZnT3 mediated the induction of COX-2 expression by ZnSO_4_. Then, high COX-2 expression increases the phosphorylation of tau by producing PGI_2_ and F_2α_ through the PI3-K/AKT, ERK1/2 and JNK/c-Jun pathways. Consistent with the pivotal roles of COX-2 and PGs in regulating tau phosphorylation, treatment with the COX-2-specific inhibitor NS398 decreases the cognitive decline of Tau^P301S^ Tg mice (Figure 9).

COX-2 is tightly regulated under physiological conditions. As an inducible enzyme, COX-2 mRNA and protein synthesis can be induced in numerous cell types, including neurons, in a time- and dose-dependent manner by metal ions, cytokines, oncogenes and growth factors [33-38]. It is thus presumed that COX-2 is primarily responsible for the elevated production of PGs at the sites of disease. Consistent with this hypothesis, Montine *et al.* [31] reported that COX-2 and PGE_2_ were upregulated during the early stage of AD. These observations indicate that the early stage of AD must include stimulators that result in the upregulation of COX-2. Among various stimulators for COX-2 expression, we found that Zn^2+^ and TNF-α can enhance the expression of COX-2. In line with our observation, Wu *et al.* [39]reported that Zn^2+^ exposure resulted in pronounced increases in COX-2 mRNA and protein expression in human airway epithelial cells. Further, Choi *et al.* [40] reported that Zn^2+^ chelation inhibits the expression of COX-2 in ischemia-activated retinal neurons. Mechanistically, Choi *et al.* [40] reported that the p38- and epithelial growth factor (EGF) receptor-activated PI3-K/AKT pathway is necessary for Zn^2+^-induced COX-2 expression. In contrast to these observations, Taccioli *et al.* [41] reported that Zn^2+^ replenishment decreases the overexpression of COX-2 in Zn^2+^ deficient rats. However, this study should be interpreted with caution because their experiments do not focus on neurons.

Apart from Zn^2+^, TNF-α also increases the expression of COX-2. In line with our observation, Tyagi *et al.* [42] reported that TNF-α can induce the expression of COX-2 in tumorigenic mouse lung epithelial LM2 cells. In addition, Kuwano *et al.* [43] reported that TNF-α enhances the production of COX-2 protein in HUVECs. These observations were further validated in human NCI-H292 epithelial cells, suggesting that TNF-α induced COX-2 expression via the phospholipase C-γ2, protein kinase C-α, tyrosine kinase, NF-κB-inducing kinase, and IκB1/2 pathways [44]. Further, Medeiros *et al.* [6] showed that TNF-α mediated the effects of Aβ_1-40_ in upregulating the expression of COX-2, which results in cognitive decline in mice. Reciprocally, Xing *et al.* [45] reported the involvement of COX-2 in hypoxia-induced TNF-α expression in osteoblasts. However, they report that TNF-α is not involved in regulating COX-2 expression in osteoblasts [45]. Nevertheless, this discrepancy might be due to cell-specific responses to TNF-α treatment. Along these lines and with our data, we can conclude that TNF-α is responsible for upregulating the expression of COX-2 during the course of AD development.

COX-2 exhibits multiple biological functions and is generally thought to regulate the pathogenesis of AD *via* its metabolic products, including PGE_2_, PGD_2_ [and its dehydration end product 15-deoxy-∆^12,14^-PGJ_2_ (15d-PGJ_2_)], PGI_2_, PGF_2α_ and TXA_2_ [46]. For example, 15d-PGJ_2_ treatment increases the cleavage of tau protein and results in the formation of ∆tau, which accelerates the formation of NFTs in human neuroblastoma SK-N-SH cells [5]. Although COX-2 inhibition or overexpression has been indicated to be involved in tau phosphorylation or the formation of NFTs [3, 4], to the best of our knowledge, there is no further evidence showing a relationship between PGs and tau phosphorylation. Therefore, we first investigated whether PGI_2_ and F_2α_ had the ability to induce the phosphorylation of tau. Consistent with this hypothesis, we found that PGI_2_ and F_2α_ could increase the phosphorylation of tau at the sites of Ser202 and Ser 400/Thr 403/Ser 404.

In view of these novel findings, we further found that the PI3-K/AKT, ERK1/2 and JNK/c-Jun pathways mediated the effects of PGI_2_ and F_2α_ in stimulating the phosphorylation of tau at both Ser 202 and Ser 400/Thr 403/Ser 404. Consistent with our findings, Baki *et al.* [47] reported that the PI3-K/AKT pathway mediated the effects of PS1 in stimulating the phosphorylation of tau via a glycogen synthase kinase-3 (GSK-3)-dependent mechanism. In addition, tau phosphorylation was also regulated by PI3-K/AKT-mediated inactivation of GSK-3β in EphB2-stimulated tau Tg mice [48]. Apart from the PI3-K/AKT pathway, hyperphosphorylation of tau is also mediated by ERK in neuroblastoma SK-N-SH cells [49]. Similarly, Carlyle *et al.* [50] reported that increased cAMP/PKA signaling is responsible for upregulating the phosphorylation of tau, which increases the risk of degeneration in aging association cortex. In addition, JNK plays a key role in tau hyperphosphorylation in AD models [51]. Consistent with these reports, our data revealed the involvement of the PI3-K/AKT, ERK1/2 and JNK/c-Jun pathways in augmenting the phosphorylation of tau.

In conclusion, this study provides new evidence for the synergistic roles of PGI_2_ and F_2α_ in regulating the phosphorylation of tau both *in vitro* and *in vivo*. Specifically, Zn^2+^ stimulated the expression of TNF-α, highly expressed TNF-α induced the expression of COX-2 and the production of PGI_2_ and F_2α_. High accumulation of PGI_2_ and F_2α_ induces the phosphorylation of tau at both Ser 202 and Ser 400/Thr 403/Ser 404 via the PI3-K/AKT, ERK1/2 and JNK/c-Jun pathways. These observations provide new insights into the mechanisms of tau phosphorylation in a PGI_2_- and F_2α_-dependent mechanism in the brain during AD development.

## MATERIALS AND METHODS

### Reagents

ZnSO_4_, PGI_2_, PGF_2α_ and the inhibitors including NS398, U0126, PD98059, LY294002 and SP600125 were obtained from Sigma-Aldrich Corp (St. Louis, MO, USA). Antibodies against β-actin, ERK1/2, p-ERK1/2, c-Jun, p-c-Jun, AKT, p-AKT, COX-2, TNF-α, p-tau (Ser 202), p-tau (Ser400/Thr403/Ser404) and tau were purchased from Cell Signaling Technology, Inc. (Danvers, MA, USA). ZnT3 antibody was obtained from Santa Cruz Biotechnology (Santa Cruz, CA, USA). TNF-α was purchased from Raybiotech, Inc. (Norcross, GA, USA). All reagents for the qRT-PCR and SDS-PAGE experiments were purchased from Bio-Rad Laboratories. All other reagents were from Invitrogen (Carlsbad, CA, USA) unless otherwise specified.

### Cell culture

Mouse neuroblastoma 2a (n2a) cells were grown (37 °C and 5% CO_2_) on 6-cm tissue culture dishes (10^6^ cells per dish) in appropriate medium. In a separate set of experiments, the cells were grown in serum-free medium for an additional 12 h before incubation with inhibitors in the absence or presence of PGI_2_ or PGF_2α_, as previously described [52].

### Transgenic mice and treatments

The wild type (WT) or Tau^P301S^ Tg mice [B6;C3-Tg(Prnp-MAPT*P301S)PS19Vle/J (Stock Number: 008169)] were obtained from The Jackson laboratory (Bar Harbor, ME, USA). Genotyping was performed at 3-4 weeks after birth. The mice were housed in a controlled environment under a standard room temperature, relative humidity and 12-h light/dark cycle with free access to food and water. Mice were randomly separated into several groups and each group contains 12 mice. Mice at 6 months of age were injected (i.c.v) with PGI_2_ (1 µg/5 µl), PGF_2α_ (1 µg/5 µl), ZnSO4 (10 µg/5 µl), TNF-α (1 ng/5 µl), or NS398 (1 µg/5 µl) for 24 h before determining the phosphorylation of tau. In select experiments, Tau^P301S^ Tg mice at the age of 1-months-old were treated with NS398 (1 mg/kg/d) for 5 months before determining the learning ability by nest construction test. The general health and body weights of animals were monitored every day. The brains of animals from the different groups were collected under anesthesia and perfusion as previously described [53-56].

### Real-Time PCR

qRT-PCR assays were performed with the MiniOpticon Real-Time PCR detection system (Bio-Rad) using total RNA and the GoTaq one-step Real-Time PCR kit with SYBR green (Promega) and the appropriate primers as previously described [57-59]. The GenBank accession number and forward and reverse primers for mouse COX-2 and GAPDH are provided in our previous publications [57]: mouse ZnT3 (NM_011773) F-GTCTCCCTCTGGATAGTCACTGG, R-GCATACTCTGCACCTGTAGATCC; TNF-α (NM_013693) F-cagaaaagcaagcagccaac, R-gggaacttctcatccctttg. The gene expression values were normalized to those of GAPDH.

### Western blots

Tissues or cells were lysed in radio-immune precipitation assay buffer (25 mM Tris-HCl [pH 7.6], 150 mM NaCl, 1% NP-40, 1% sodium deoxycholate, and 0.1% SDS) containing protease inhibitor cocktail (Pierce Chemical Company). The protein content of the cell lysates was determined using a bicinchoninic acid (BCA) protein assay reagent (Pierce Chemical Company). The total cell lysates (4 μg) were subjected to SDS-PAGE, transferred to a membrane, and probed with a panel of specific antibodies. Each membrane was only probed with one antibody. β-actin was used as a loading control. All western hybridizations were performed at least in triplicate using a different cell preparation each time.

### Intracerebroventricular injection

NS398, PGI_2_, PGF_2α_, ZnSO_4_, TNF-α or vehicle (PBS) were injected (i.c.v) to WT or Tau^P301S^ Tg mice as previously described [53-56]. Briefly, stereotaxic injections were placed at the following coordinates from the bregma: mediolateral: -1.0 mm; anteroposterior: -0.22 mm; dorsoventral: -2.8 mm. Following injection, each mouse recovered spontaneously on a heated pad. The reliability of injection sites were validated by injecting trypan blue dye (Invitrogen) in separate cohorts of mice and observing staining in the cerebral ventricles. Twenty-four hours after injection, mice were harvested after anaesthesia and perfusion [53-56].

### Immunofluorescence

Brain tissues were collected from WT or Tau^P301S^ Tg mice. Serial sections (10-µM thick) were cut by cryostats (Leica, CM1850, Germany). After fixation with 4% paraformaldehyde, slides were stained with p-Tau (Ser 202), p-Tau (Ser 400/Thr 403/Ser 404), TNF-α, COX-2 or ZnT3 antibody with Alexa Fluor 555 secondary antibodies (Cell Signaling Technology, Inc., Danvers, MA, USA) or Alexa Fluor 488 secondary antibodies (Jackson ImmunoResearch, Inc., West Grove, PA, USA) before observing under confocal microscopy (Leica, TCS-SP8, Leica).

### Nest construction

Nest construction experiments were performed as previously described [56]. One week after corn cob nest-building test, the corn cob was replaced with clean ones. Two hours prior to the onset of the dark phase of the lighting cycle, 8 pieces of paper (5×5 cm^2^) were introduced in the home cage to create conditions for nesting. The nests were scored the following morning along a 4-point system: (1) no biting/rearing with random dispersion of the paper, (2) no biting/tearing of paper with gathering in a corner/side of the cage, (3) moderate biting/bearing on paper with gathering in a corner/side of the cage, and (4) extensive biting/tearing on paper with gathering in a corner/side of the cage.

### Animal committee

All animals were handled according to the care and use of medical laboratory animals (Ministry of Health, Peoples Republic of China, 1998) and all experimental protocols were approved by the Laboratory Ethics Committees of College of Life and Health Sciences of Northeastern University.

### Human brain samples

Human brain samples were obtained from New York Brain Bank, serial numbers TT4263 (early stage of AD, the patient is 73-years-old man who was diagnosed as a mild AD patient), T4308 (middle stage of AD, the patient is 86-years-old man who was diagnosed as moderate AD patient), T4339 and T4304 (late stage of AD, the patients are 88-years-old woman and 84 years-old woman who were diagnosed as severe and end stage of AD patients).

### Statistical analysis

All data are represented as the mean ± S.E. of at least three independent experiments. The statistical significance of the differences between the means was determined either using Student’s *t*-test [52-56].

## SUPPLEMENTARY MATERIALS FIGURES


