# Supplementary material for: COX-2 metabolic products, the prostaglandin I2 and F2α, mediate the effects of TNF-α and Zn2+ in stimulating the phosphorylation of Tau
Source: Oncotarget. 2017 Oct 16;8(59):99296–311. doi: 10.18632/oncotarget.21853 (PMC5725093; doi:10.18632/oncotarget.21853)
Supplement: Supplementary file 1 [file oncotarget-08-99296-s001.pdf]

COX-2 metabolic products, the prostaglandin I_2 and $F_{2\alpha}$, mediate the effects of TNF-α and Zn^{2+} in stimulating the phosphorylation of Tau

SUPPLEMENTARY MATERIALS

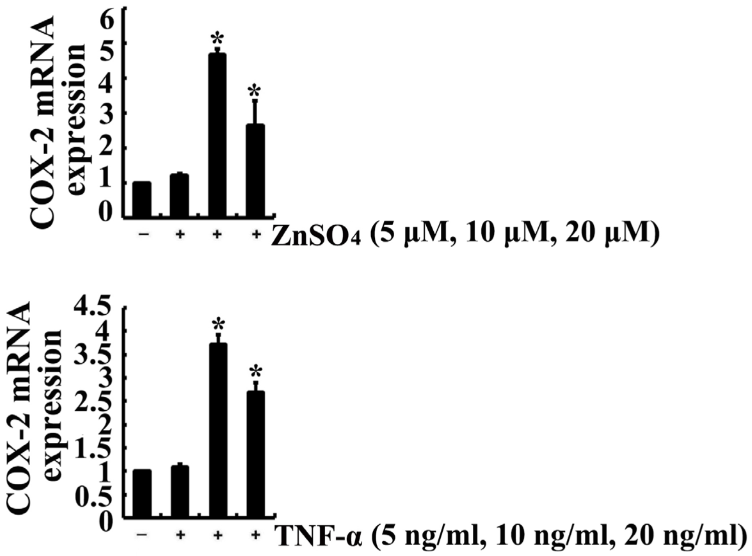

Supplementary Figure 1:

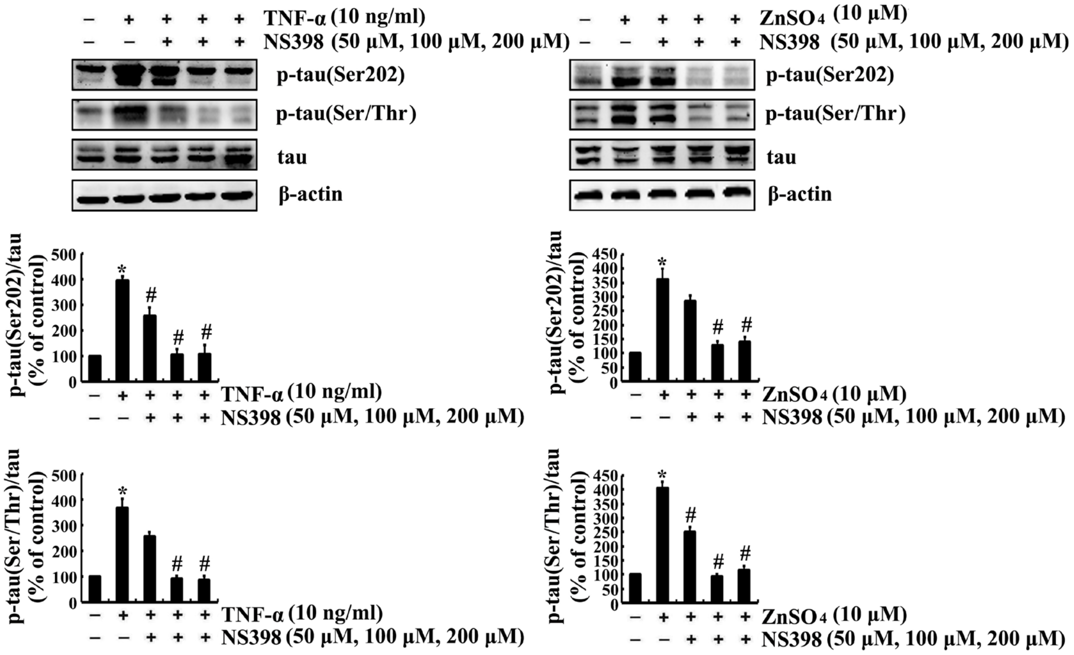

Supplementary Figure 2:

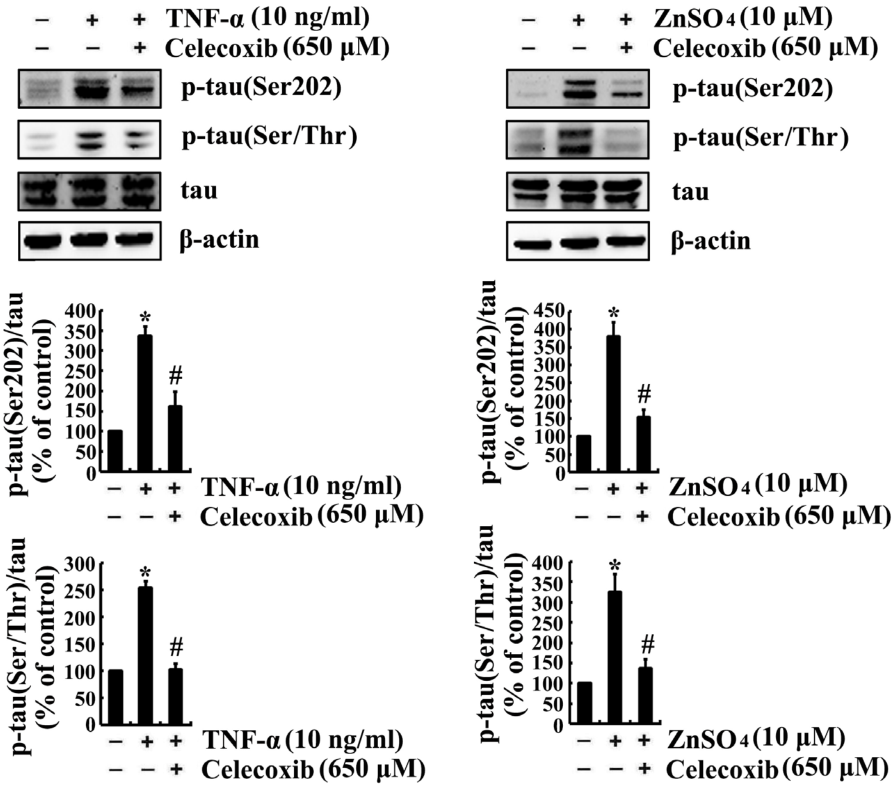

Supplementary Figure 3:
